# Verification of the Cross Immunoreactivity of A60, a Mouse Monoclonal Antibody against Neuronal Nuclear Protein

**DOI:** 10.3389/fnana.2016.00054

**Published:** 2016-05-13

**Authors:** Shanping Mao, Guoxiang Xiong, Lei Zhang, Huimin Dong, Baohui Liu, Noam A. Cohen, Akiva S. Cohen

**Affiliations:** ^1^Department of Neurology, Renmin Hospital, Wuhan UniversityWuhan, China; ^2^Department of Anesthesiology and Critical Care Medicine, Children’s Hospital of Philadelphia, University of PennslyvaniaPhiladelphia, PA, USA; ^3^Philadelphia Veterans Affairs Medical Center, University of PennslyvaniaPhiladelphia, PA, USA; ^4^Departments of Otorhinolaryngology—Head and Neck Surgery, University of PennslyvaniaPhiladelphia, PA, USA; ^5^Department of Anesthesiology and Critical Care Medicine, Perelman School of Medicine, University of PennslyvaniaPhiladelphia, PA, USA

**Keywords:** hybridoma, brain sections, neurocytoma, stem cell differentiation, immunohistology, epitopes

## Abstract

A60, the mouse monoclonal antibody against the neuronal nuclear protein (NeuN), is the most widely used neuronal marker in neuroscience research and neuropathological assays. Previous studies identified fragments of A60-immunoprecipitated protein as Synapsin I (Syn I), suggesting the antibody will demonstrate cross immunoreactivity. However, the likelihood of cross reactivity has never been verified by immunohistochemical techniques. Using our established tissue processing and immunofluorescent staining protocols, we found that A60 consistently labeled mossy fiber terminals in hippocampal area CA3. These A60-positive mossy fiber terminals could also be labeled by Syn I antibody. After treating brain slices with saponin in order to better preserve various membrane and/or vesicular proteins for immunostaining, we observed that A60 could also label additional synapses in various brain areas. Therefore, we used A60 together with a rabbit monoclonal NeuN antibody to confirm the existence of this cross reactivity. We showed that the putative band positive for A60 and Syn I could not be detected by the rabbit anti-NeuN in Western blotting. As efficient as Millipore A60 to recognize neuronal nuclei, the rabbit NeuN antibody demonstrated no labeling of synaptic structures in immunofluorescent staining. The present study successfully verified the cross reactivity present in immunohistochemistry, cautioning that A60 may not be the ideal biomarker to verify neuronal identity due to its cross immunoreactivity. In contrast, the rabbit monoclonal NeuN antibody used in this study may be a better candidate to substitute for A60.

## Introduction

Neuronal nuclear protein (NeuN) has been characterized as a neuron-specific nuclear deoxyribonucleic acid (DNA)-binding regulatory molecule (Mullen et al., 1992). Using repeated immunization with purified nuclei from neurons, a mouse monoclonal antibody was generated and named A60 (Mullen et al., 1992). The mouse monoclonal antibody is capable of labeling neurons out of mitotic stages in different species of vertebrates (Mullen et al., 1992; Rodriguez et al., 2002; Tonchev et al., 2003; Kumar and Buckmaster, 2007; Korzhevskii et al., 2009; Verdiev et al., 2009). However, A60 has shown a lack of labeling for cerebellar Purkinje cells, olfactory mitral cells and retinal photoreceptor cells (Mullen et al., 1992). Further neuronal exceptions may include cerebellar interneurons (Weyer and Schilling, 2003) and a subset of substantia nigral neurons (Weyer and Schilling, 2003; Kumar and Buckmaster, 2007; Cannon and Greenamyre, 2009; Korzhevskii et al., 2009; Verdiev et al., 2009).Highly specific for labeling postmitotic neurons, A60 has been most widely used neuronal marker in neuroscience research and neuropathological assays (Gusel’nikova and Korzhevskiy, 2015). The spectrum of A60 use is very broad including labeling to monitor neuronal development (Wynder et al., 2005), neurogenesis (Magavi et al., 2000) and stem cell differentiation (Brazelton et al., 2000). NeuN imunoreactivity can also be used as a biomarker for diagnosis of central neurocytoma (Wolf et al., 1996). Furthermore, studies have suggested that quantitative changes in NeuN immunoreactivity can be a determinant of neuronal loss in several pathologies including neurodegenerative diseases (Tippett et al., 2007), ischemia (Korzhevskii et al., 2009), axotomy (McPhail et al., 2004) and traumatic brain injury (Igarashi et al., 2001). However, Unal-Cevik et al. (2004) has cautioned that loss of NeuN immunoreactivity after cerebral ischemia might not directly indicate neuronal cell loss. That is, it is likely that some pathologies will alter the antigen-antibody interaction. For example, altering NeuN protein phosphorylation leads to diminished reactivity with A60 (Lind et al., 2005).NeuN is a soluble nuclear protein, delineated as two to three bands at 46–48 kilo-dalton (kD) as demonstrated by Western blotting (Mullen et al., 1992). These bands have been identified as Fox-3 protein, a member of Fox-1 gene family of splicing factors (Kim et al., 2009). Furthermore, immunoprecipitation with Millipore A60 pulled down two additional protein bands near 70 kD (Kim et al., 2009; Maxeiner et al., 2014). The proteins pulled down with A60 were identified as Synapsin I (Syn I) using mass spectrometry; thereby, suggesting cross reactivity of the NeuN antibody. Surprisingly, this cross immunoreactivity between A60 and Syn I could only be observed using Western blotting and was never observed using standard immunohistochemical staining in paraffin-embedded brain slices (Kim et al., 2009; Maxeiner et al., 2014). Here we succeeded in revealing A60 cross immunoreactivity by processing brain tissues differently and performing free floating immunofluorescent staining in vibratome prepared slices. We also used a rabbit monoclonal antibody against NeuN to verify the cross reactivity.

## Materials and Methods

Six to eight week old male mice (C57/Bl6, Jackson Laboratory, Bar Harbor, ME, USA) were used. The procedures and protocols for all animal studies were approved by Institutional Animal Care and Use Committees of Wuhan University, Children’s Hospital of Philadelphia and University of Pennsylvania, in accordance with international guidelines on the ethical use of animals (National Research Council, 1996). All immunostaining and Western blotting was undertaken in three animals in order to verify a distribution pattern.

### Immunofluorescent Staining

For all immunofluorescent experiments, we used our own published protocols (Xiong et al., 2012, 2015; Yuan et al., 2015). For free floating immunofluorscent staining, 15 mice were deeply anesthetized with 0.4 ml of 5% chloral hydrate and perfused with normal saline followed by 4% paraformaldehyde in 0.1 M phosphate buffer (pH 7.4). Brains were removed and post-fixed for 90 min at room temperature (RT). Brains were kept in PBS at 4°C before frontal slices (50 μm thickness) were cut with a Leica VT 1000s vibratome (Leica Microsystems Inc., Buffalo Grove, IL, USA). To minimize the number of animals sacrificed, brain slices were collected in six series for different immunostaining settings with an interval of 300 μm between two adjacent slices within an identical series (Xiong et al., 2012). The slices were permeabilized with 0.3% Triton X-100 and blocked with a mixture of 5% normal goat serum and 1% bovine serum albumin at RT for 60 min, respectively.

For single immunofluorescent staining, slices were incubated with a mouse or rabbit antibody for 90 min at RT followed by overnight at 4°C. For visualization, we used Alexa Fluor 488-conjugated goat anti-mouse IgG or Alexa Fluor 594-conjugated goat anti-rabbit IgG (Both at 1:250 in PBS; Life Technology, Grand Island, NY, USA) at RT for 60 min. The nuclear dye Hoechst (Life Technology, Grand Island, NY, USA) was added to the secondary antibody solution in order to counterstain the samples. For double staining, we incubated the brain slices with a mouse and a rabbit antibody simultaneously. For visualization, a mixture of Alexa Fluor 488-conjugated goat anti-mouse IgG (green) and Alexa Fluor 594-conjugated goat anti-rabbit IgG (rabbit) were used, together with Hoechst (blue). Procurement and working dilution for the antibodies are listed in Table 1. For imaging with Olympus Fluoview 1000 system (Olympus America, Center Valley, PA, USA), we applied the same confocal settings as previously published (Xiong et al., 2012). Brain structure nomenclature was derived from Paxinos and Franklin (2001).

**Table 1:** **List of antibodies used in the present study**.

Antigen name (abbreviation)	Manufacture	Cat. #	Host	Immunogen	Original concentration	Dilution for immunostaining	Dilution for western blot
Neuronal nuclear protein (NeuN)	Zymed (South San Francisco, CA, USA)	18–7373 (Discontinued)	Mouse Mono (A60)	Mouse brain nuclei	100 μg/ml	1:500	N/A
NeuN	Millipore (Billerica, MA, USA)	MAB377	Mouse Mono (A60)	Mouse brain nuclei	1000 μg/ml	1:250	1:500
NeuN	Abcam (Cambridge, MA, USA)	ab177487	Rabbit Mono	Human NeuN aa 1–100	727 μg/ml	1:500	1:1000
Synapsin I (Syn I)	Sigma (St. Louis, MO, USA)	S193	Rabbit Poly	Bovine Synapsin I	200 μg/ml	1:500	1:1000
Vesicular glutamate transporter 1 (VGLUT1)	Synaptic Systems (Goettingen, Germany)	135 302	Rabbit Poly	Rat VGLUT1 aa 456–560	serum	1:2000	N/A
Vesicular GABA transporter (VGAT)	Synaptic Systems	131 002	Rabbit Poly	Rat GABA aa 75–87	serum	1:250	N/A
Glial fibrillary acidic protein (GFAP)	Abcam	ab48050	Rabbit Poly	Human GFAP aa 1–400	1000 μg/ml	1:1000	N/A

*Major information from manufactures and working dilution of each antibody were listed. Mono, monoclonal antibody; Poly, polyclonal antibody*.

### Western Blot

Three mice were anesthetized with isoflurane and subsequently decapitated. The entire protocol for protein extraction was performed on ice. The brains were removed and submerged in cold saline. Each hippocampus (HC) was immediately dissected out and chopped into three pieces. The hippocampal tissue blocks were collected in lysis buffer containing 50 mM Tris, 1mM EDTA, 150 mM NaCl, 1% Triton X-100, 1% sodium deoxycholate, 0.1% SDS and 1% protease inhibitor (Sigma-Aldrich, St. Louis, MO, USA). As a control, cortical tissue blocks were also collected into a separate tube. After gentle homogenization with a pellet pestle, the homogenate was sonicated at a level set at 15 for 20 s (5 s-on/3 s-off) with a sonic dismembrator (Model 500, Fisher Scientific, Pittsburgh, PA, USA). The resulting homogenate was centrifuged at 15,000 g for 20 min. and the supernatant was collected. Protein concentration was determined and the samples were aliquoted and stored at −80°C for subsequent Western blotting.

Double fluorescent western blotting was performed after staining patterns of target proteins was confirmed with single blots. Equal amounts (by weight) of hippocampal or cortical protein samples were separated with SDS-PAGE gel (7.5%) electrophoresis and transferred to nitrocellulose membranes. The membranes were blocked in PBST buffer (0.1% tween 20 in PBS) with 5% non-fat milk at RT for 60 min. They were subsequently incubated with either a mixture of Millipore A60 and rabbit anti-NeuN, or Millipore A60 and rabbit anti-Syn I in non-fat milk at 4°C overnight. After washing thoroughly in PBST, the membranes were incubated with a mixture of Alexa Fluor 800 conjugated goat anti-mouse IgG (green) and Alexa Fluor 680 conjugated goat anti-rabbit IgG (red) at RT for 60 min. Both secondary antibodies (Rockland Immunochemicals Inc., Pottstown, PA, USA) were used at a 1:10,000 dilution in non-fat milk. The fluorescent immunoblots were imaged with Odyssey imaging system (Li-Cor Biosciences, Lincoln, NE, USA).

## Results

### “Unexpected” Synaptic Staining by Millipore A60

**Figure 1:**
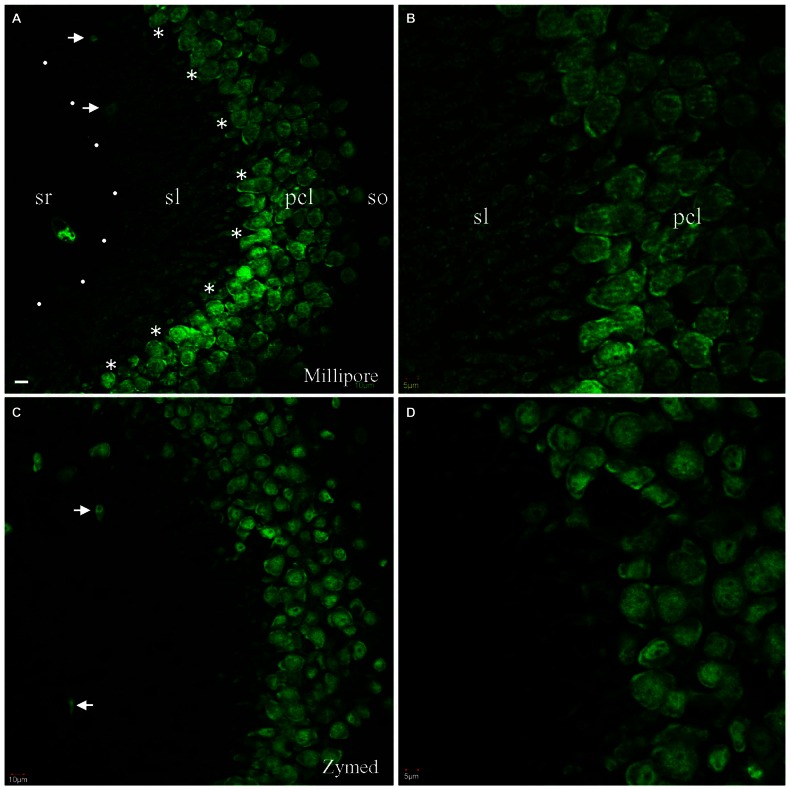
**Comparison of Millipore A60 to Zymed A60 in immunofluorescent staining pattern.** Confocal images from hippocampal area CA3ab subregion. A60 from either Millipore **(A,B)** or Zymed **(C,D)** intensively labeled nuclei and somata of principal cells and inhibitory interneurons (*arrows*). Truncated proximal neuropils were prominent adjacent to pyramidal cell layer (pcl). In addition to this common staining pattern, Millipore A60 also resulted in punctate staining in stratum lucidum (sl), delineated by *dots* and *asterisks ***(A)**. so, stratum oriens; sr, stratum radiatum. Scale bar: 10 μm in **(A,C)**; 5 μm in **(B,D)**.

To counterstain neuronal populations in the HC, cortex (CTX) and other brain areas, we combined either the Zymed or Millipore A60 antibody with additional antibodies for target molecules. Neuronal nuclei as well as cell bodies were heavily stained by both A60 antibodies, leaving nucleoli unstained (Figure 1). Occasionally the proximal neurites were also labeled, which often occurred in the CTX. Glia and blood cells/vessels never demonstrated any labeling. Unexpectedly, Millipore A60 also labeled large sized punctate structures in hippocampal area CA3, in addition to transversely truncated neurites adjacent to pyramidal cell layer. These punctate structures could be clearly identified even under low power magnification and were distributed exclusively in stratum lucidum (sl) from CA3c to CA3ab (Figures 1A,B), suggesting that they might be mossy fiber terminals. The identical synaptic labeling pattern was confirmed using different lots of Millipore A60. In contrast, Zymed A60 did not label mossy fiber-like structures (Figures 1C,D).

### Synaptic Staining Confirmed with Millipore A60 and Synaptic Markers

**Figure 2:**
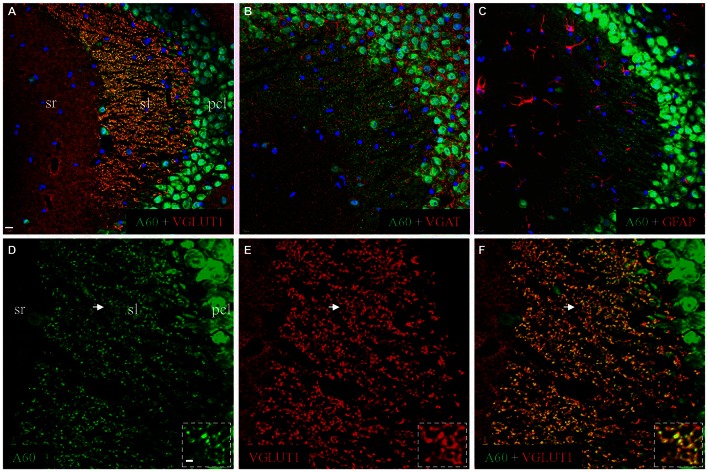
**Millipore A60-positive punctate structures might be hippocampal mossy fiber terminals.** Confocal images from CA3ab after double immunofluorescent staining with Millipore A60 and different markers. When double stained with Millipore A60 and VGLUT1, costaining could be clearly identified in punctate structures in sl (**A**, yellow). These Millipore A60-positive puncta were negative to VGAT **(B)** or GFAP **(C)**. Under higher magnification **(D–F)**, rosette-shaped structures were intensively stained by both Millipore A60 and VGLUT1, showing a perfect localization (*Insets*). Arrows indicate rosettes magnified. Scale bar: 10 μm in **(A–C)**; 5 μm in **(D–F)**; 2 μm in *Insets*.

To reveal the identity of A60-positive punctate structures in sl, we performed double immunofluorescent staining by combining the Millipore A60 with a combination of different markers (Figure 2). A perfect co-localization was demonstrated by co-staining with VGLUT1 (Figures 2A,D–F), but not with VGAT (Figure 2B) or GFAP (Figure 2C). As shown in high magnification (Figure 2, ***Inset***), these A60-positive puncta (green) displayed rosette shapes ranging from 2 to 6 μm and could be co-stained with VGLUT1 (red), suggesting that they were mossy fiber terminal (Xiong et al., 2012).

### Immunofluorescent Co-Staining and Double Fluorescent Western Blotting with Millipore A60 and Syn I Antibody

**Figure 3:**
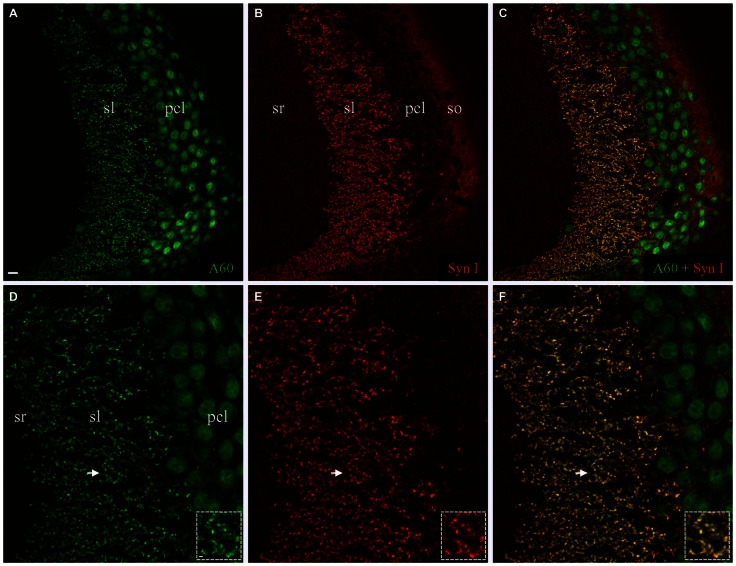
**Colocalization of Synapsin I (Syn I) in Millipore A60-positive mossy fiber terminals. (A–C)** Stacked confocal images showing distribution pattern of Millipore A60 **(A)** and Syn I **(B)** staining in CA3ab. In addition to large-sized mossy fiber terminals in sl **(C)**, Syn I-positive small puncta were also visible in sr, pcl and so **(B,C)**. **(D–F)** Confocal images under higher magnification showing perfect colocalization (yellow) in mossy fiber terminals. *Arrows* indicating rosette-shaped mossy fiber terminals highlighted in *Insets*. Scale bar: 10 μm in **(A–C)**; 5 μm in **(D–F)**; 2 μm in *Insets*.

We also performed co-staining of Millipore A60 with a rabbit polyclonal antibody against Syn I (Figure 3). The pan-synaptic marker Syn I resulted in intensive immunostaining in sl and weak labeling in other hippocampal subregions (Figures 3B,E). In sl where mossy fiber terminals were intensively stained by A60 (Figures 3A,D), a perfect colocalization was evident (Figures 3C,F, ***Insets***).

**Figure 4:**
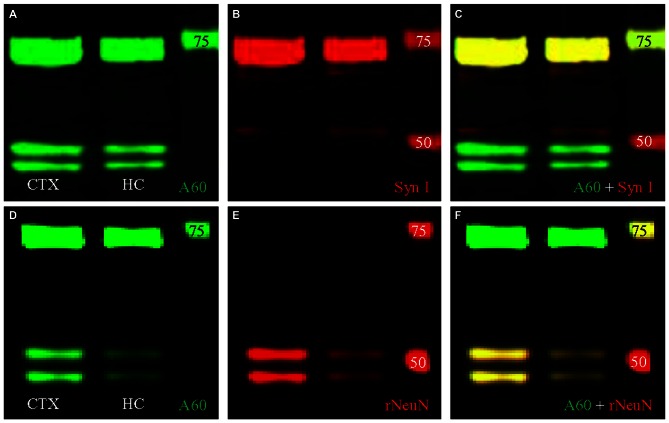
**Double fluorescent Western blotting using Millipore A60 and rabbit antibody against Syn I or NeuN. (A–C)** Millipore A60 detected two bands around 50 kilo-dalton (kD) and a single wide band close to 75 kD **(A)** in both hippocampus (HC) and cortex (CTX), while Syn I antibody intensively stained a wide band near 75 kD **(B)**. Perfect co-staining was clearly identified in the band near 75 kD **(C)**. **(D–F)** The rabbit anti-NeuN recognized two bands around 50 kD **(E)** and co-staining by both NeuN antibodies was identified in these two bands **(F)**, leaving the band near 75 kD positive for Millipore A60 only **(D,F)**. Arabic numerals indicate the size (in kD) of markers.

We then conducted double fluorescent Western blotting with both antibodies using protein samples extracted from the HC, to determine if the bands close to 74 kD could be labeled by both Millipore A60 and Syn I, as reported previously (Kim et al., 2009; Maxeiner et al., 2014). Our Western blotting protocol using Millipore A60 labeled two bands at approximately 50 kD and another wide band at approximately 75 kD (Figure 4A). Syn I labeling showed an intensively stained wide band near 75 kD (Figure 4B). A perfect co-staining was clearly identified in the band at approximately 75 kD (Figure 4C, yellow), leaving two bands around 50 kD exclusively labeled by A60 only. Surprisingly, the co-stained band near 75 kD was also apparent in protein samples from the CTX (Figure 4).

### Saponin Treatment Improved Synaptic Staining by Millipore A60

**Figure 5:**
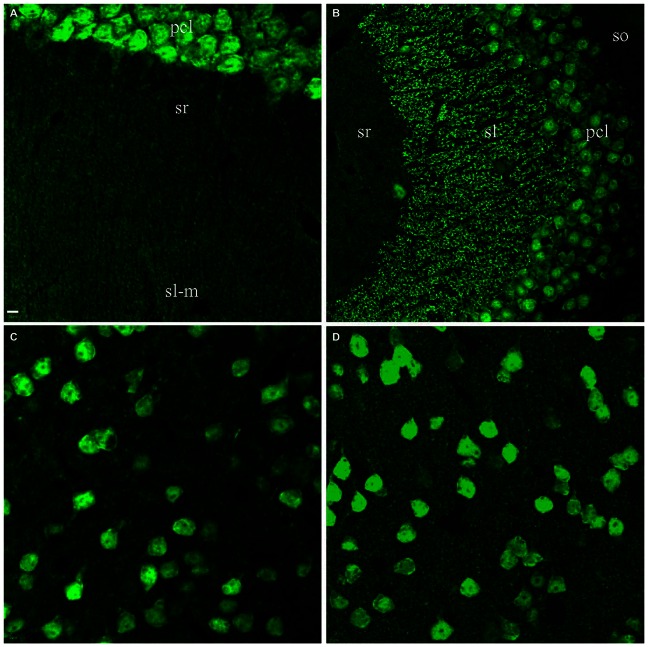
**Synaptic staining from Millipore A60 could be improved by saponin treatment.** Stacked confocal images showing immunostaining pattern. Hippocampal area CA1 was the only region showing clear punctate staining of small synapses (0.5–1 μm) in slices treated with Triton X-100 **(A)**. **(B)** Clear staining of small synapses in sr **(B)** of area CA3 after saponin treatment. Compared to treatment with Triton X-100 **(C)**, saponin treatment also improved synaptic staining from Millipore A60 in the CTX **(D)**. Scale bar: 5 μm in **(A,C,D)**; 10 μm in **(B)**.

The identification of the co-stained 75 kD band from cortical samples lead us to assess A60 synaptic staining carefully in the whole brain using high power magnification. In addition to mossy fibers in sl of area CA3, hippocampal area CA1 was the only further area demonstrating A60-positive punctate staining, ranging between 0.5 to 1 μm (Figure 5A). These A60-positive small synaptic structures were mainly distributed in stratum radiatum and/or lacunosum-moleculare, likely along pyramidal neuronal dendritic arbors. In the CTX as well as other brain regions, no synaptic staining by Millipore A60 was evident (Figure 5C).

Considering that other synaptic structures might not be well preserved for Millipore A60 immunostaining under our standard protocol, we replaced Triton X-100 with saponin in order to keep more vesicular proteins prior to immunostaining as previously reported (Goldenthal et al., 1985). Compared to the Triton X-100 treated brain slices (Figures 1–3), saponin treatment enhanced synaptic staining by Millipore A60 in the HC (Figure 5B), CTX (Figures 5C,D), thalamus and other areas of the brain (data not shown).

### Double Fluorescent Western Blot and Immunofluorescent Co-Staining with Millipore A60 and a Rabbit Monoclonal Antibody Against NeuN

**Figure 6:**
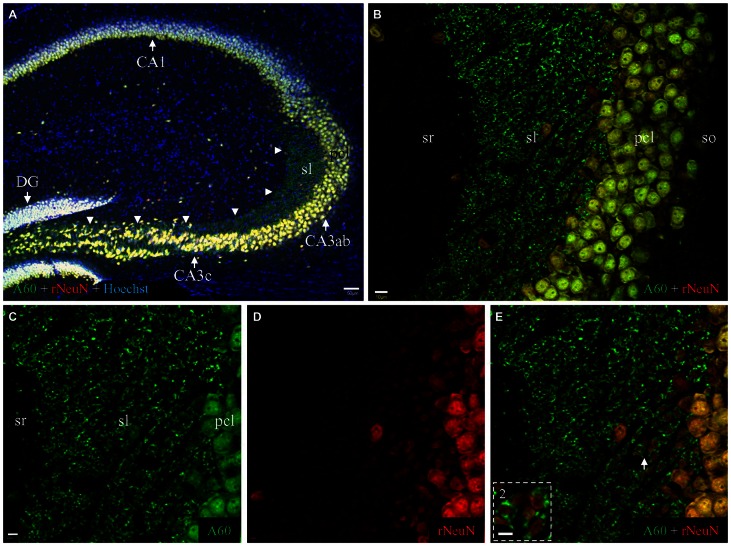
**Comparison of Millipore A60 (green) to the rabbit anti-NeuN (red) by double immunofluorescent costaining. (A)** Staining pattern in the whole HC. Principal cells and interneurons were intensively stained by both antibodies, exhibiting white or off-white appearance due to the addition of counterstaining with Hoechst (blue). Punctate staining was prominent along mossy fiber pathway (*arrowheads*), stained by Millipore A60 only (green). DG, dentate gyrus. **(B)** Stacked confocal images showing distribution pattern stained by both NeuN antibody in CA3ab. Whereas double stained neuronal nuclei and cell bodies (yellow) were prominent in pcl, mossy fiber terminals were positive to Millipore A60 only (green), highlighted in sl. **(C–E)** Single confocal image under higher magnification showing double stained mossy fiber terminals. (*Inset*) Rosette-shaped mossy fiber terminals encircling truncated neuropils to form sheath-like arrangements, highlighted from the area indicated by *Arrow*. Scale bar: 50 μm in **(A)**; 10 μm in **(B)**; 5 μm in **(C–E)**; 2 μm in *Inset*.

To verify the cross immunoreactivity, we performed double fluorescent Western blotting (Figures 4D–F) as well as immunofluorescent co-staining (Figure 6) with Millipore A60 and a rabbit monoclonal antibody against NeuN. The rabbit monoclonal anti-NeuN labeled two bands at approximately 50 kD (Figure 4E) in both CTX and HC. These bands were perfectly matched with the bands labeled by A60 (Figure 4F). However, the band at approximately 75 kD was labeled with Millipore A60 (Figure 4D) but no labeling was evident with the rabbit anti-NeuN (Figures 4E,F).

Double immunohistochemical labeling demonstrated that the rabbit anti-NeuN specifically labeled neuronal nuclei/cell bodies as efficiently as Millipore A60 (Figure 6A, white or off-white). No punctate structures were stained by the rabbit anti-NeuN, thus mossy fiber terminals were solely labeled by Millipore A60 only (Figure 6A, ***arrowheads***) evident as pure green puncta (Figures 6B–E). The rabbit anti-NeuN antibody labeled more truncated neuropils of hippocampal principal neurons (Figure 6D), perhaps due to the lack of synaptic staining in the area. Sheath-like arrangements (Figure 6E, ***Inset***) of A60 positive mossy fiber terminals (green) were prominent, surrounding truncated neuropils (orange to yellow).

**Figure 7:**
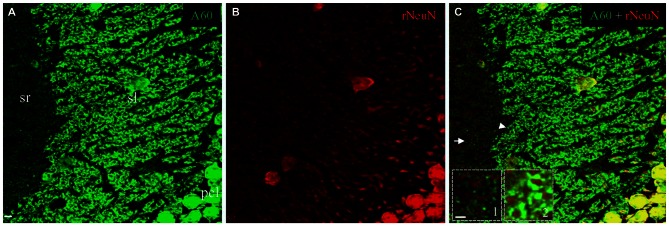
**No synaptic staining could be achieved by the rabbit anti-NeuN after saponin treatment.** Single confocal image taken from area CA3ab showing double staining with Millipore A60 and the rabbit anti-NeuN. Whereas Millipore A60 (green) stained out mossy fiber terminals in sl and small synapses in sr **(A)**, truncated neuropils stained by the rabbit NeuN antibody (red) were prominent in both proximal and distal sl **(B)**. Colocalization (yellow) could be seen exclusively in nuclei and somata of principal cells and inhibitory interneurons **(C)**. No staining by the rabbit anti-NeuN was found either in small synapses from sr (*Inset 1*), or in mossy fiber terminals from sl (*Inset 2*). *Arrow* shows the area of small synapses in sr. *Arrowhead* indicates mossy fiber terminals forming sheath-like arrangements, even to distal sl. Scale bar: 5 μm in **(A–C)**; 2 μm in *Insets*.

To test if triton treatment might diminish the rabbit anti-NeuN synaptic labeling as occurred with the Millipore A60 antibody, we once again conducted double immunostaining in brain slices treated with saponin. In contrast to Millipore A60-positive synaptic staining (green) in sl and sr (Figure 7A), no synaptic staining by the rabbit NeuN antibody (red) was evident in either subregion (Figure 7B). Furthermore, no co-staining could be identified in synaptic structures (Figures 7A–C, ***Insets***), except for double labeled neuronal cell bodies/nuclei (Figure 7C). Compared to treatment with Triton X-100 (Figure 6D), saponin treatment demonstrated more truncated neuropiles by the rabbit anti-NeuN (Figure 7B), resulting more sheath-like arrangements, even in the distal sl (Figure 7, ***Inset*** **2**).

## Discussion

Our established immunofluorescent staining protocol applied in vibratome prepared brain slices (Xiong et al., 2012, 2015; Yuan et al., 2015), was used to counterstain neuronal cell groups with Zymed A60, a monoclonal antibody against NeuN. This antibody intensively labeled nuclei and cell bodies of principal neurons and inhibitory interneurons in similar fashion to that reported by Mullen et al. (1992), without nonspecific labeling of glia, blood cells or vessels. Since the Zymed A60 antibody had been discontinued, we necessarily needed to use the Millipore A60 antibody. The Millipore monoclonal resulted in a similar staining pattern of neuronal cell bodies and nuclei. However, we encountered “unexpected” labeling of punctate structures in sl of hippocampal area CA3, which is known as the termination target of hippocampal mossy fibers (Amaral and Witter, 2000). Based on the size (2–6 μm), location (sl), morphology (rosette-shape) and immunoreactivity (positive for VGLUT1), these Millipore A60-positive punctate structures were thought to be mossy fiber terminals (Amaral and Witter, 2000; Xiong et al., 2012). The present study demonstrated that the two A60 antibodies from different sources s might give rise to different labeling patterns, even though both antibodies were generated from same hybridoma clone originally developed by Mullen et al. (1992). Furthremore, due to the unavailability of the Zymed A60 antibody, we could not determine the source of the differences in synaptic staining.

Previous Western blotting with the Millipore A60 antibody resulted in two protein bands at approximately 74 kD (Kim et al., 2009; Maxeiner et al., 2014). Using mass spectrometry these bands has been identified as Syn I, suggesting that A60 cross reacts with the Syn I protein. The authors (Kim et al., 2009) had to use whole cell extracts from neurons (but not the original immunogen, i. e. purified neuronal nuclear extracts) to detect the putative Syn I bands, confirming that the target protein (i.e., Syn I) involved in the cross reactivity was not a nuclear component. In the present study we show a single band of similar size, possibly due to a lower separating efficiency of our SDS-Page gel electrophoresis. This band could be labeled with both Syn I antibody and A60, supporting the notion of A60 cross reactivity (Kim et al., 2009; Maxeiner et al., 2014). The present and previous (Kim et al., 2009; Maxeiner et al., 2014) data obtained with Western blotting led us to perform double immunofluorescent labeling. Interestingly, we found a perfect co-localization of Syn I in Millipore A60-positive mossy fiber terminals, contradicting previous reports that cross reactivity has not been supported by immunohistochemical experimentation conducted in fixed brains (Kim et al., 2009; Maxeiner et al., 2014; Gusel’nikova and Korzhevskiy, 2015).

The quality of immunohistochemical staining may be largely dependent on fixation, tissue processing/sectioning, permeabilizing reagents, and the quality and concentration of the primary antibody (Werner et al., 2000; Bussolati and Leonardo, 2008; Shi et al., 2008; Fung and Tam, 2010; Xiong et al., 2015; Yuan et al., 2015). We note that previous studies investigating possible A60 cross reactivity were performed on paraffin-embedded slices (Kim et al., 2009; Maxeiner et al., 2014). Further, it has been suggested that the Syn I protein might be vulnerable to the necessary tissue processing for paraffin embedding including heating, alcoholic dehydration and xylene dewaxing (Maxeiner et al., 2014). Moreover, over fixation of brain tissue might be the key factor attributing to the negative staining of synaptic structures. Unlike 90 min used in the current study for post-fixation in paraformaldehyde (Xiong et al., 2012, 2015; Yuan et al., 2015), previous studies post-fixed brains for 12 h overnight or even up to 48 h (Kim et al., 2009; Maxeiner et al., 2014). It has previously demonstrated that aldehyde fixatives can cross-link specific amino acid residues and thus alter a protein’s quaternary structure (Puchtler and Meloan, 1985; Metz et al., 2004; Toews et al., 2008). Therefore, excessive fixation might result in muffling the epitopes of the target proteins. Coincidently, the core epitope in Syn I for A60 reaction is more likely to be modified by aldehyde fixation (Maxeiner et al., 2014). It is worthy to mention that our Syn I labeling demonstrated both mossy fiber terminals and small-sized synapses in triton-treated slices (Figure 3). However, labeling with Millipore A60 did not demonstrate small synapses in the same slices, indirectly supporting the notion put forth by Kim et al. (2009) that A60 has a lower affinity for Syn I protein.

Like the pan-presynaptic marker synaptophysin (Xiong et al., 2012), Syn I should be expressed all over the brain, suggesting that the cross immunoreactivity of A60 should exist in other brain regions in addition to the HC. Western blotting in cortical samples demonstrated co-staining with Millipore A60 and Syn I antibodies in the 75 kD band. However, our synaptic staining by Millipore A60 could only be observed within the HC. This inconsistency might be due to a yet to be identified factor(s) muffling other regions from immunostaining. It has been suggested that Triton X-100 use leads to artificial loss of some antigens and therefore saponin might be a better detergent for preparation for immunohistochemical labeling of membranous or vesicular proteins (Goldenthal et al., 1985). The use of Triton X-100 might diminish Syn I cross reactivity with Millipore A60 in most synapses, except for hippocampal mossy fiber terminals (with large volume) and small-sized synapses along CA1 pyramidal cell dendrite trees (with high density). Interestingly, saponin use led to robust labeling of small-sized synapses all over the brain including HC, CTX, thalamus as well as other regions of the brain. However, saponin might not be strong enough to permeate the thick slices used in the present study, because the labeling all occurred within a short range from brain slice surfaces (data not shown).

Technically, when neuronal nuclei (around 5 μm) and cell bodies were in focus, tiny synapses (less than 1 μm) might be easily ignored unless rigorously examined with high power objectives. Due to the large size, Millipore A60-positive mossy fiber terminals could be easily identified as a curved band along CA3c to CA3ab, even under low power objectives (Figure 6A). In fact, A60-stained small synapses in area CA1 could not be clearly identified until examined with 63× objective.

To definitively verify antibody cross immunoreactivity, one of the following suggestions should be followed. Negative staining with gene knockout samples or co-staining with two antibodies raised from different animal species and designed to recognize different epitopes of the target protein (Lorincz and Nusser, 2008; Saper, 2009; Xiong et al., 2015). Since it is impossible to generate a knockout NeuN animal due to its biological importance (Dredge and Jensen, 2011; Kim et al., 2011, 2013), we followed the second suggestion using the mouse monoclonal (Millipore A60) immunized by purified neuronal nuclei together with a rabbit monoclonal antibody immunized by synthetic peptide of human NeuN protein. We demonstrated that synaptic staining could be identified by Millipore A60 but not by the rabbit anti-NeuN in slices treated with or without saponin, suggesting that the cross reactivity might come from the epitope corresponding to the 75 kD band (Syn I) rather than approximately 50 kD bands (Fox-1). The verification of the cross reactivity of Millipore A60 was also supported by our Western blotting data demonstrating the Millipore A60-positive band near 75 kD could not be stained by the rabbit anti-NeuN. It has been a long-term quandary that A60 cross reactivity could be confirmed by immunochemical but not immunohistological techniques (Kim et al., 2009; Maxeiner et al., 2014; Gusel’nikova and Korzhevskiy, 2015). The present study sheds some interesting evidence on this issue.

In summary, Millipore A60 should not be solely used to prove neuronal identity due to the potential cross immunoreactivity demonstrated in the present and previous studies (Kim et al., 2009; Maxeiner et al., 2014). Without similar cross reactivity, the rabbit monoclonal NeuN antibody tested here may be better suited than A60. Alternatively, a careful verification with an additional NeuN antibody is necessary when attempting to interpret immunostaining labeling with Millipore A60.

## Author Contributions

SM: collected, analyzed and interpreted data; revised the manuscript. GX: designed the study; collected, analyzed and interpreted data; edited the manuscript. LZ, HD and BL: collected and analyzed data; revised the manuscript. ASC and NAC: designed the study; revised the manuscript. All authors approved the final version and agreed to be accountable for all aspects of the work.

## Funding

The present study was supported by NIH grants R37-HD059288 and R01-NS069629 (to ASC).

## Conflict of Interest Statement

The authors declare that the research was conducted in the absence of any commercial or financial relationships that could be construed as a potential conflict of interest.
